# A universal equation-of-state model based on single variable functions

**DOI:** 10.1038/s41598-025-93314-9

**Published:** 2025-03-12

**Authors:** Ti-Wei Xue, Zeng-Yuan Guo

**Affiliations:** Key Laboratory for Thermal Science and Power Engineering of Ministry of Education, Department of Engineering Mechanics, Tsinghua University, Beijing, 100084 China

**Keywords:** Equation of state, Ideal gas, Thermodynamics, Single variable function, Thermodynamics, Applied physics

## Abstract

Since the ideal gas equation of state (EOS) was established in 1840, a wide variety of EOS theories have been developed. However, due to the diversity of material structures and the complexity of intermolecular interactions, numerous EOS either have complex forms or have empirical coefficients without physical meaning, which severely limits their applications. This paper builds a simple and universal EOS model by means of a fully macroscopic thermodynamic approach. Firstly, two single variable thermodynamic functions as a function of pressure only and as a function of temperature only, respectively, are constructed. On this basis, two EOS in the forms of *P–V–T* and *P–S–T* are obtained by thermodynamic derivation, which are almost as simple as the ideal gas EOS. There are no assumptions about material structures and intermolecular interactions involved here. Therefore, the model is universal. Moreover, the coefficients in these two EOS have clear thermodynamic significance and thus can be calculated directly without fitting. The model is shown to characterize the thermodynamic properties of substances well and may play an important role in high-density and supercritical applications. This work may provide a new way of developing EOS theory and enrich the fundamentals of thermodynamics.

## Introduction

A simple and universal equation of state (EOS) is a rich source for research and discussion. Such an EOS usually expresses the commonality of thermodynamic properties of matter. The ideal gas EOS is the best known of these EOS:

1 $$PV = RT.$$

It shows a simple property that all substances share in the low-density limit^1^. The ideal gas EOS, because of its simplicity and universality, has become the most widely discussed and applied EOS. Early in 1873, on the basis of the ideal gas model, the famous van der Waals EOS was established by considering the intermolecular attraction and the volume occupied by the molecules themselves^2,3^. Since then, the extrapolation based on the ideal gas limit has become the most common approach to developing an EOS for individual substances. For example, the virial EOS^4^, the Redlich-Kwong EOS^5^, the Peng-Robinson EOS^6^ and the Benedict-Webb-Rubin EOS^7,8^ are representative of this type of EOS. However, for high-density matter far from the ideal gas state, the approach of extrapolation suffers a dilemma due to complicated and diverse molecular interactions^9–15^. Instead, a great number of empirical EOS with complicated forms or a large number of coefficients have been established^16–20^. Since these empirical EOS cover only specific states or individual substances, when they are extrapolated beyond their range fitted to experimental data, the results are commonly unreliable^21–23^.

Recently, there has been great progress on this issue. An ideal dense matter EOS symmetric to the ideal gas EOS was proposed by means of von Oettingen’s symmetric exchanges of variables, *T* ↔ *P* and -*S* ↔ *V*^24^:

2 $$TS = R^{\prime}P,$$

where $R^{\prime}$ is another physical constant related to the thermodynamic properties of matter, which was called “*the ideal dense matter constant*”. It is symmetric to the ideal gas constant, *R*, and can be considered as the limiting slope of isobaric expansion coefficient at infinite pressure that can be obtained by extrapolation from experiments^24^. Thermodynamic symmetry implies that the ideal dense matter EOS is a high-density limiting model and all matter should share this simple relation at ultra-high densities. The verification work from both experiments and simulations has shown that the higher the density (higher pressure or lower temperature) of matter, the more accurate the description of the ideal dense matter EOS^24,25^. It has also been verified that the interpolation based on the ideal dense matter model can well characterize the explosions and the shock-Hugoniots^26^. Different from the method of modifying the ideal gas model by taking into account the structures and interactions of individual substances, the establishment of the ideal dense matter EOS employed a fully macroscopic thermodynamic approach. As a result, the ideal dense matter model is independent of any assumption on material structure and therefore universal. It successfully extracted the common features of thermodynamic properties of high-density substances.

Although both the ideal gas model and the ideal dense matter model are extremely simple in form, they, as limiting models, cannot be used to characterize the intermediate density state well without some modifications. In this paper, a universal EOS model for intermediate densities is derived by again adopting a fully macroscopic thermodynamic approach. It consists of two EOS, *P–V–T* and *P–S–T*, whose forms are as simple as the ideal gas EOS or the ideal dense matter EOS. Due to being independent of any assumption on material structure, this universal model reflects the common features of thermodynamic properties at intermediate densities. It is shown to agree well with the property data of substances. This work may provide a new perspective for understanding the thermodynamic properties of matter and developing EOS theory.

## Single variable functions

### Dimensionless coefficients

It is well known that a distinctive feature of the ideal gas model is that the internal energy is a function of temperature only, *U* = *U* (*T*), expressed in differential form as

3 $${\text{d}}U = C_{V} {\text{d}}T.$$

As a comparison, it has been demonstrated that the internal energy of the ideal dense matter model is a function of the pressure only, *U* = *U* (*P*), whose differential form is^24,25^

4 $${\text{d}}U = C_{S} {\text{d}}P,$$

where *C*_*S*_ is the specific work (capacity) at constant entropy, defined as^24,27,28^.

5 $$C_{S} = \left( {\frac{\partial U}{{\partial P}}} \right)_{S} { = } - P\left( {\frac{\partial V}{{\partial P}}} \right)_{S} .$$

It represents the amount of work input to the system from the external environment per unit of pressure increase under isentropic conditions. von Oettingen^29^ argued that *C*_*S*_ was the physical quantity symmetric to the specific heat (capacity) at constant volume, *C*_*V*_:

6 $$C_{V} = \left( {\frac{\partial U}{{\partial T}}} \right)_{V} = T\left( {\frac{\partial S}{{\partial T}}} \right)_{V} .$$

For the low-density limit, *U* is a function of *T* only and for the high-density limit, *U* is a function of *P* only. For intermediate density states, *U* is jointly determined by both *T* and *P*, i.e., *U* = *U* (*T*, *P*). In order to develop an intermediate-density EOS model that is expected to have the same simple form as the ideal gas EOS or the ideal dense matter EOS, the following assumptions are made. That is, for intermediate-density states, there exists a certain thermodynamic function that depends on temperature only, and also a certain thermodynamic function that depends on pressure only. These two single variable functions can be obtained by modifying internal energy, which is inspired by the thermodynamic definition of the Grüneisen coefficient, *γ*^30^:

7 $$\gamma = V\left( {\frac{\partial P}{{\partial U}}} \right)_{V} = \left( {\frac{\partial PV}{{\partial U}}} \right)_{V} .$$

From a perspective of macroscopic thermodynamics, the Grüneisen coefficient expresses the ratio of the energy function, *PV*, to the internal energy, *U*, under isochoric conditions. Experience has shown that the Grüneisen coefficient can generally be regarded as a constant or as a function of volume only^31^:

8 $$\gamma = {\text{Const}}\;{\text{or}}\;\gamma \left( V \right).$$

A new thermodynamic function, *E*_*γ*_, can be defined as

9 $$E_{\gamma } = U - \frac{1}{\gamma }PV.$$

Based on Eqs. (7) and (8), it can be proved that *E*_*γ*_ is a function of volume only:

10 $$E_{\gamma } = E_{\gamma } \left( V \right).$$

Replacing *P* and *V* with each other in the definition of *γ* yields a new dimensionless coefficient, *α*:

11 $$\alpha = \left( {\frac{\partial PV}{{\partial U}}} \right)_{P} .$$

Another new dimensionless coefficient, *β*, is further obtained based on *α* by means of von Oettingen’s symmetric exchanges of variables, *T* ↔ *P* and -*S* ↔ *V*:

12 $$\beta = - \left( {\frac{\partial TS}{{\partial U}}} \right)_{T} .$$

*α* expresses the ratio of the energy function, *PV*, to the internal energy, *U*, under isobaric conditions and *β* expresses the ratio of the energy function, *TS*, to the internal energy, *U*, under isothermal conditions. The two new coefficients, *α* and *β*, are defined analogously to the Grüneisen coefficient, *γ*. These three coefficients can be regarded as functions of a same family. According to the definitions, the following thermodynamic relation for *α* can be obtained:

13 $$\alpha = \frac{1}{{\frac{1}{PV}\frac{{C_{P} }}{{\alpha_{P} }} - 1}},$$

and the following thermodynamic relation for *β* can be obtained:

14 $$\beta = \frac{1}{{\frac{P}{T}\frac{{\beta_{T} }}{{\alpha_{P} }} - 1}},$$

where *α*_*P*_ is the isobaric expansion coefficient, defined as

15 $$\alpha_{P} = \frac{1}{V}\left( {\frac{\partial V}{{\partial T}}} \right)_{P} ,$$

and *β*_*T*_ is the isothermal compression coefficient, defined as

16 $$\beta_{T} = - \frac{1}{V}\left( {\frac{\partial V}{{\partial P}}} \right)_{T} .$$

**Fig. 1:**
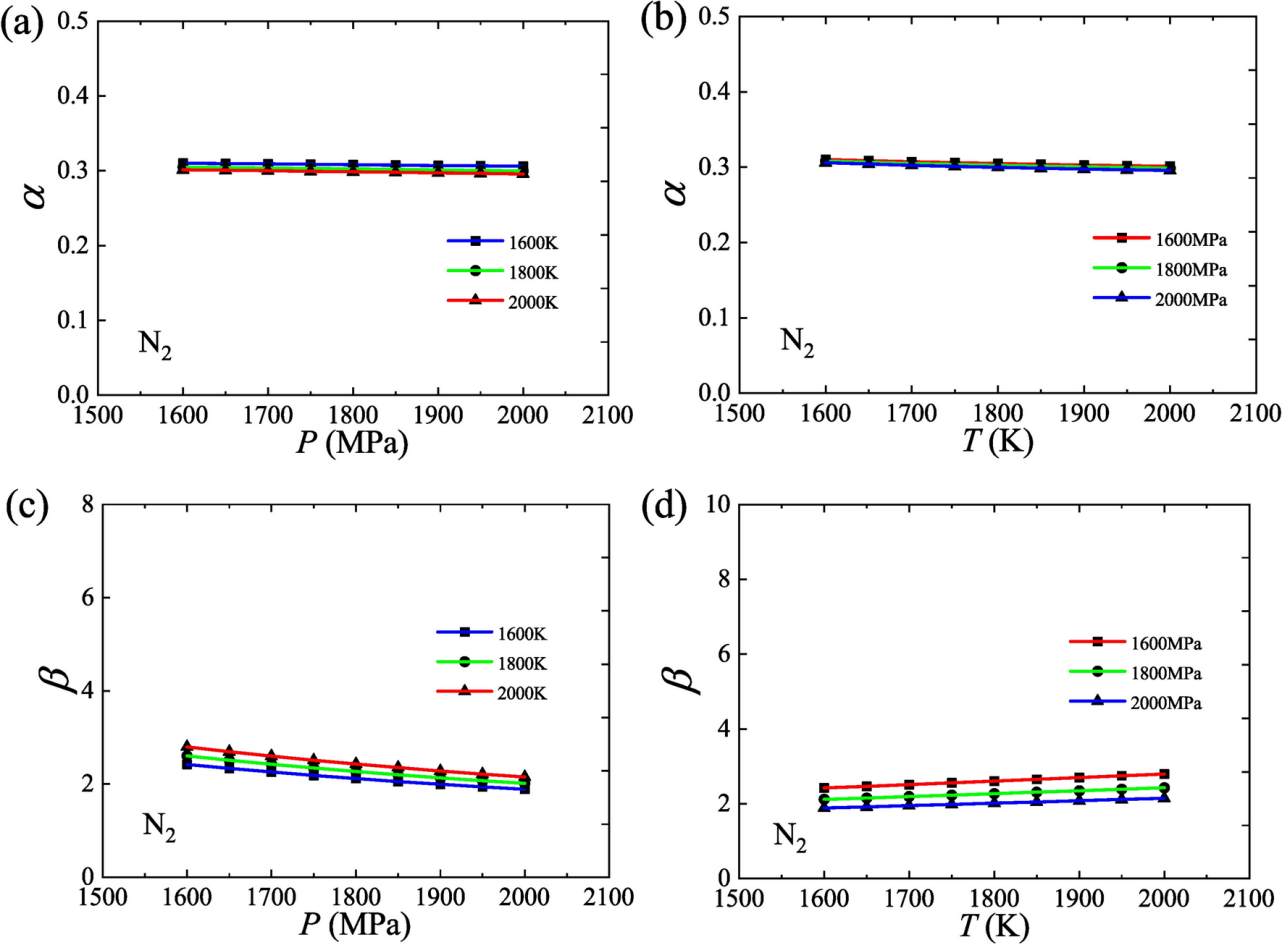
The calculated values of these two new coefficients, *α* and *β*, for N_2_. (**a**) Isotherms, *α* versus *P*; (**b**) Isobars, *α* versus *T*; (**c**) Isotherms, *β* versus *P*; (**d**) Isobars, *β* versus *T*.

The values of *α* and *β* for N_2_ are calculated based on Eqs. (13) and (14), where the values of *C*_*P*_, *α*_*P*_, and *β*_*T*_, are from the National Institute of Standards and Technology (NIST). Both *α* and *β*, in particular *α*, have a good property of being constant over the selected variation range (1600–2000 K, 1600–2000 MPa), where *α* take a value near 0.3 and *β* take a value near 2, as shown in Fig. 1. As with the Grüneisen coefficient, both *α* and *β* are weak functions of pressure or temperature and, therefore, given a proper variation range of matter states, they can be regarded as constants.

### Single variable functions

Define a new thermodynamic function, *E*_*α*_, based on Eq. (11):

17 $$E_{\alpha } { = }U - \frac{1}{\alpha }PV.$$

It can be proved that when *α* is regarded as a constant, the thermodynamic function is a function of the pressure only, *E*_*α*_ = *E*_*α*_ (*P*), whose differential form is

18 $${\text{d}}E_{\alpha } = C_{\alpha } {\text{d}}P,$$

where *C*_*α*_ is the derivative of the thermodynamic function, *E*_*α*_, with respect to pressure and has the physical connotation of “*work capacity*”. With entropy and pressure as independent variables, the expression for *C*_*α*_ is obtained based on Eqs. (17) and (18):

19 $$C_{\alpha } { = }\left( {1 + \frac{1}{\alpha }} \right)C_{S} - \frac{1}{\alpha }V.$$

Similarly, define another new thermodynamic function, *E*_*β*_, based on Eq. (12):

20 $$E_{\beta } { = }U{ + }\frac{1}{\beta }TS.$$

When *β* is regarded as a constant, the thermodynamic function is a function of the pressure only, *E*_*β*_ = *E*_*β*_ (*T*), whose differential form is

21 $${\text{d}}E_{\beta } = C_{\beta } {\text{d}}T,$$

where *C*_*β*_ is the derivative of the thermodynamic function, *E*_*β*_, with respect to temperature and has the physical connotation of “*heat capacity*”. With volume and temperature as independent variables, the expression for *C*_*β*_ is obtained based on Eqs. (20) and (21):

22 $$C_{\beta } { = }\left( {1 + \frac{1}{\beta }} \right)C_{V} + \frac{1}{\beta }S.$$

**Fig. 2:**
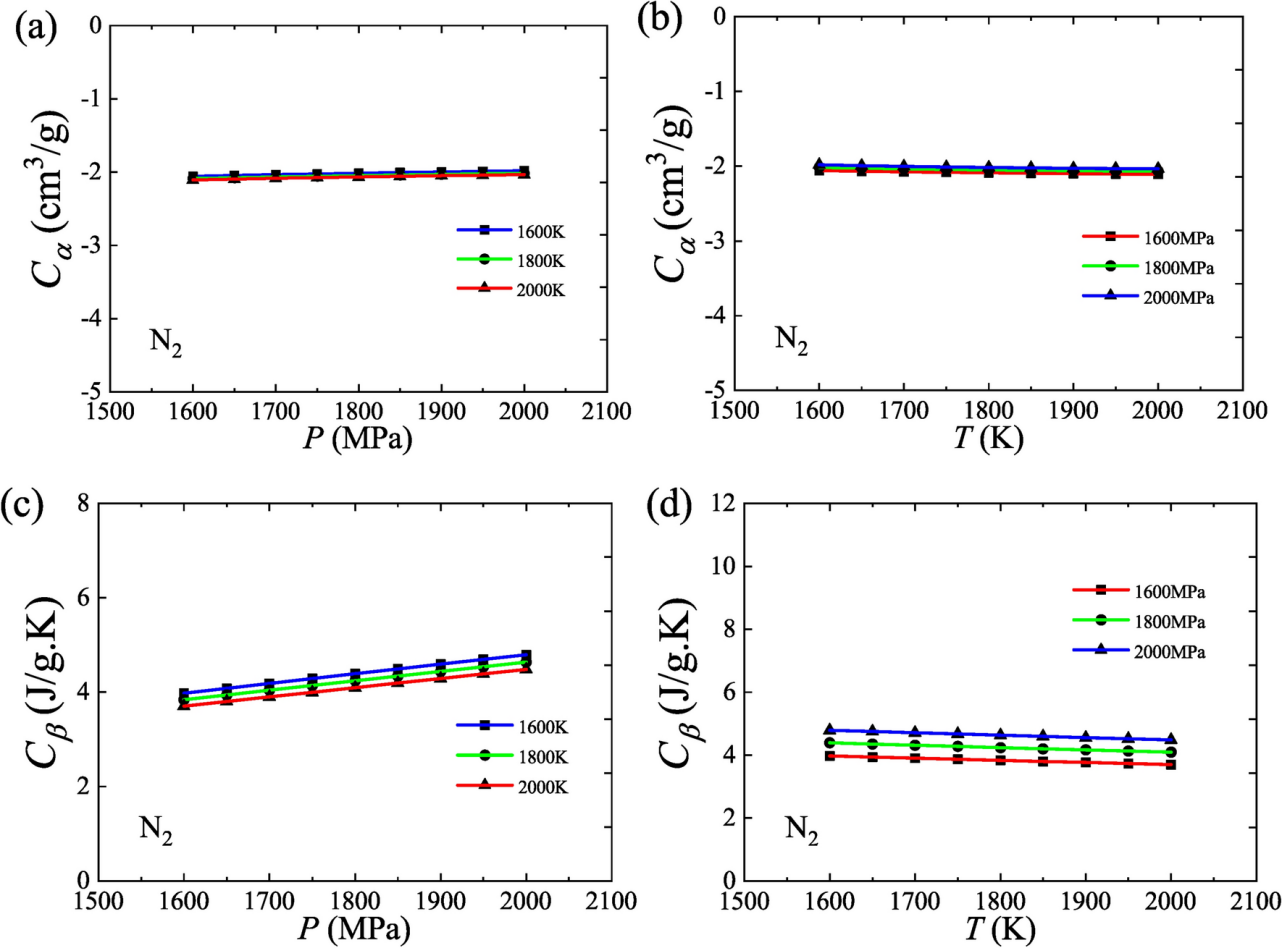
The calculated values of *C*_*α*_ and *C*_*β*_ for N_2_. (**a**) Isotherms, *C*_*α*_ versus *P*; (**b**) Isobars, *C*_*α*_ versus *T*; (**c**) Isotherms, *C*_*β*_ versus *P*; (**d**) Isobars, *C*_*β*_ versus *T*.

The values of *C*_*α*_ and *C*_*β*_ for N_2_ are calculated using Eqs. (19) and (22), where the data are from NIST. Both *C*_*α*_ and *C*_*β*_, in particular *C*_*α*_, have a good property of being constant over the selected variation range (1600–2000 K, 1600–2000 MPa), where *C*_*α*_ take a value near −2 and *C*_*β*_ take a value near 4, as shown in Fig. 2. Both* C*_*α*_ and *C*_*β*_ are weak functions of pressure or temperature and, therefore, given a proper variation range, they can be regarded as constants.

## Modeling

When both *α* and *β* are regarded as constants, these two thermodynamic functions, *E*_*α*_ and *E*_*β*_*,* become single variable functions of pressure and temperature, respectively. When both *C*_*α*_ and *C*_*β*_ are regarded as constants, *E*_*α*_ and *E*_*β*_ have linear characteristics, respectively. Based on the above approximations or assumptions, thermodynamic derivation gives two EOS in *P–V–T* and *P–S–T* forms (See Supplementary Information):

23 $$V = \alpha CT^{{\frac{\beta }{{1 + \alpha { + }\beta }}}} P^{{ - \frac{{1{ + }\beta }}{{1 + \alpha { + }\beta }}}} - \alpha C_{\alpha } ,$$

24 $$S = - \beta CT^{{ - \frac{1 + \alpha }{{1{ + }\alpha + \beta }}}} P^{{\frac{\alpha }{{1{ + }\alpha { + }\beta }}}} { + }\beta C_{\beta } ,$$

where the constant, *C*, is the result of mathematical integration, and its physical significance is made clear later. Next, we derive the expression for the internal energy under this model. Based on Eqs. (11) and (23), the partial derivative of the internal energy with respect to temperature is obtained:

25 $$\left( {\frac{\partial U}{{\partial T}}} \right)_{P} = \frac{1}{\alpha }P\left( {\frac{\partial V}{{\partial T}}} \right)_{P} = \frac{\beta }{{1 + \alpha { + }\beta }}CT^{{ - \frac{1 + \alpha }{{1 + \alpha { + }\beta }}}} P^{{\frac{\alpha }{{1 + \alpha { + }\beta }}}} .$$

Based on Eqs. (12) and (24), the partial derivative of the internal energy with respect to pressure is obtained:

26 $$\left( {\frac{\partial U}{{\partial P}}} \right)_{T} = - \frac{1}{\beta }T\left( {\frac{\partial S}{{\partial P}}} \right)_{T} = \frac{\alpha }{{1{ + }\alpha { + }\beta }}CT^{{\frac{\beta }{{1{ + }\alpha + \beta }}}} P^{{ - \frac{1 + \beta }{{1{ + }\alpha { + }\beta }}}} .$$

Integrating Eq. (25) yields

27 $$U = CT^{{\frac{\beta }{1 + \alpha + \beta }}} P^{{\frac{\alpha }{1 + \alpha + \beta }}} + f\left( P \right).$$

where *f* (*P*) is a mathematical integral term under isobaric conditions, and as such, it is a function of pressure or a constant. For simplicity, it is written uniformly as a function of pressure here. Integrating Eq. (26) yields.

28 $$U = CT^{{\frac{\beta }{1 + \alpha + \beta }}} P^{{\frac{\alpha }{1 + \alpha + \beta }}} + f\left( T \right).$$

where *f* (*T*) is a mathematical integral term under isothermal conditions, and as such, it is a function of temperature or a constant. Both Eqs. (27) and (28) express the internal energy of the proposed model, so they should have the same form. Therefore, *f* (*P*) and *f* (*T*) can only be equal to the same constant:

29 $$f\left( P \right) = f\left( T \right) = {\text{Const}}{.}$$

Thus, the expression for the internal energy under this model is obtained:

30 $${\text{d}}U = C{\text{d}}\left( {T^{{\frac{\beta }{{1 + \alpha { + }\beta }}}} P^{{\frac{\alpha }{{1 + \alpha { + }\beta }}}} } \right).$$

When the state of matter tends to the low-density limit, $\rho \to 0$, then $\beta \to \infty$, and, accordingly, Eq. (30) is reduced to Eq. (3). At this time, the constant, *C*, becomes the specific heat (capacity) at constant volume, *C*_*V*_:

31 $$\mathop {\lim }\limits_{\rho \to 0} C = C_{V} .$$

When the state of matter tends to the high-density limit, $\rho \to \infty$, then $\alpha \to \infty$, and, accordingly, Eq. (30) is reduced to Eq. (4). At this time, the constant, *C*, becomes the specific work (capacity) at constant entropy, *C*_*S*_:

32 $$\mathop {\lim }\limits_{\rho \to \infty } C = C_{S} .$$

That is, the constant, *C*, has a physical connotation of “*capacity*” of energy. At the intermediate densities, it is a “*fusion*” of heat capacity and work capacity. At lower densities, it exhibits more heat capacity property, while at higher densities, it exhibits more work capacity property.

Further, based on Eq. (25), we get the relation between *C* and *C*_*V*_ in general case:

33 $$C_{V} = \frac{1 + \alpha + \beta }{{1 + \beta }}\left( {\frac{\partial U}{{\partial T}}} \right)_{P} = \frac{\beta }{1 + \beta }CT^{{ - \frac{1 + \alpha }{{1 + \alpha + \beta }}}} P^{{\frac{\alpha }{1 + \alpha + \beta }}} ,$$

and based on Eq. (26), the relation between *C* and *C*_*S*_:

34 $$C_{S} = \frac{1 + \alpha + \beta }{{1 + \alpha }}\left( {\frac{\partial U}{{\partial P}}} \right)_{T} = \frac{\alpha }{1 + \alpha }CT^{{\frac{\beta }{1 + \alpha + \beta }}} P^{{ - \frac{1 + \beta }{{1 + \alpha + \beta }}}} .$$

Replacing *C* in Eq. (23) with *C*_*V*_ yields a *P–V–T* EOS in a simple form:

35 $$P\left( {V - V_{0} } \right) = Z_{1} RT,$$

where

36 $$V_{0} = - \alpha C_{\alpha } ,$$

37 $$Z_{1} = \alpha \left( {1 + \frac{1}{\beta }} \right)\frac{{C_{V} }}{R}.$$

Similarly, replacing *C* in Eq. (24) with *C*_*S*_ yields a *P–S–T* EOS in a simple form:

38 $$T\left( {S - S_{0} } \right) = Z_{2} R^{\prime}P,$$

where

39 $$S_{0} = \beta C_{\beta } ,$$

40 $$Z_{2} = - \beta \left( {1 + \frac{1}{\alpha }} \right)\frac{{C_{S} }}{R^{\prime}}.$$

Both *Z*_1_ and *Z*_2_ are dimensionless combinatorial thermodynamic quantities. Equations (35) and (38) constitute the final EOS model. The forms of these two EOS are almost as simple as the ideal gas EOS or the ideal dense matter EOS. It can be demonstrated that when the state of matter tends to the low-density limit, $\rho \to 0$, then $Z_{1} \to 1$, and, accordingly, Eq. (35) is reduced to the ideal gas EOS, Eq. (1). Similarly, when the state of matter tends to the high-density limit, $\rho \to \infty$, then $Z_{2} \to 1$, and, accordingly, Eq. (38) is reduced to the ideal dense matter EOS, Eq. (2).

The proposed EOS model is derived from a purely macroscopic thermodynamic approach. It does not involve any assumptions about material structure, is not subject to any density conditions, and is therefore a universal model. The model reflects the commonalities in thermodynamic behavior of matter and may serve as a basis for modifications to determine more accurate properties of individual substances. Moreover, the coefficients in EOS have clear thermodynamic meaning and can be determined in advance from other accessible parameters, rather than having to be obtained by experimental fitting. That is, the model may be viewed to some extent as an a priori model. However, this model has its intrinsic limitations. Since it is derived from a purely macroscopic thermodynamic approach, it is not able to describe or predict microscopic interactions and molecular structural changes. Dramatic transformations of the molecular structure under high pressure are common, for example, the conversion of carbon dioxide into a glassy state^32^, which cannot be described by this model. This model also cannot cover other special regions such as regimes where spontaneous chemical reactions exist. In addition, since the thermodynamic derivation is based on the single-variable dependent characteristics (treating *α* and *β* as constants) of the thermodynamic functions, *E*_*α*_ and *E*_*β*_, and their linear characteristics (treating *C*_*α*_ and *C*_*β*_ as constants), the universal model may be restricted with respect to the variation range of matter states.

## Verifications

Although the thermodynamic derivation of EOS is independent of material structure, they need to be examined by property data of substances to show their justifiability.

**Fig. 3:**
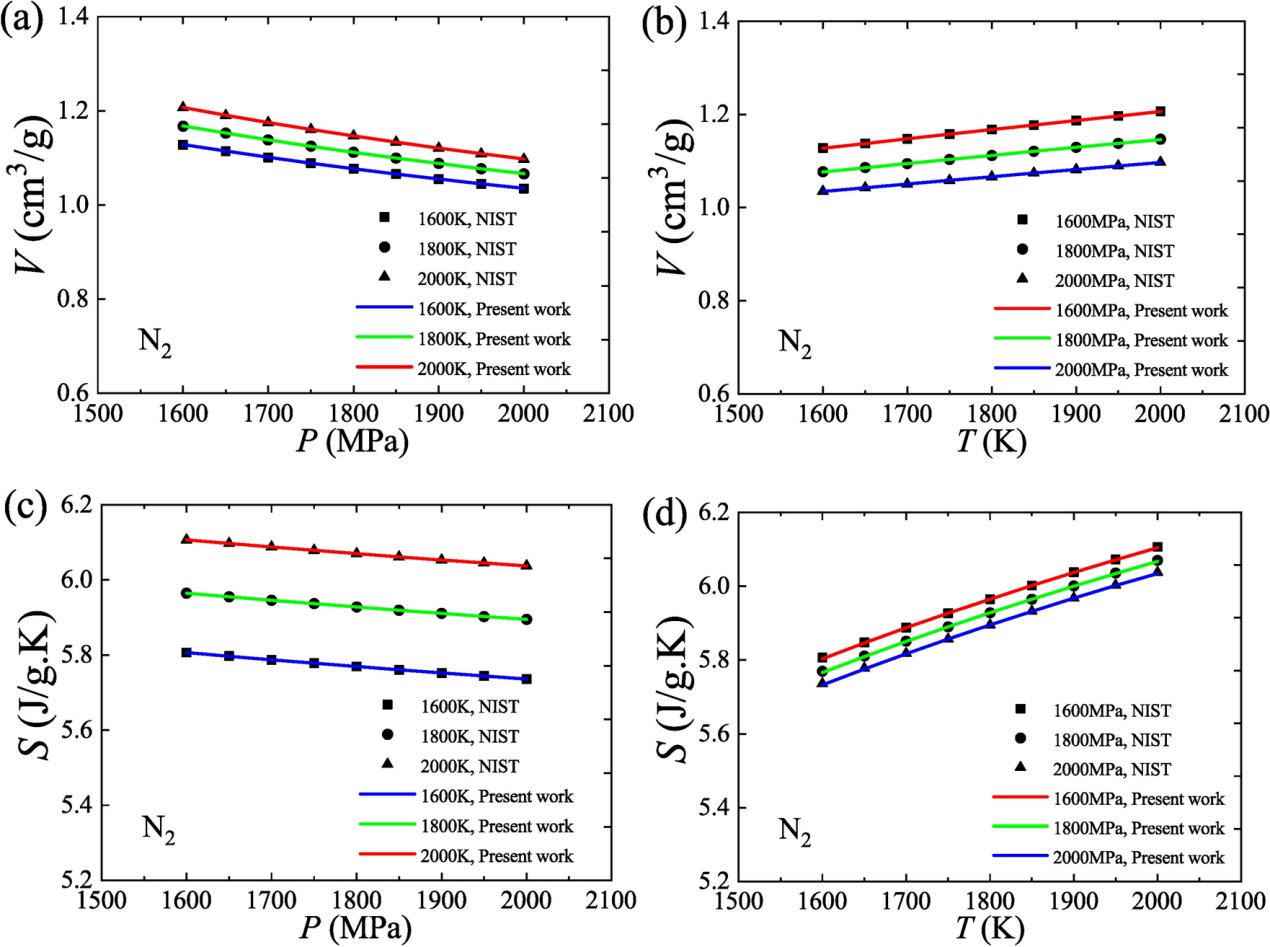
Verification of volume and entropy using N_2_ data. (**a**) Isotherms, *V* versus *P*; (**b**) Isobars, *V* versus *T*; (**c**) Isotherms, *S* versus *P*; (**d**) Isobars, *S* versus *T*.

The values of volume and entropy for N_2_ are calculated by Eqs. (35) and (38), respectively, in the absence of free-fitting parameters, where the normal boiling point (NBP) is chosen as the zero point of entropy (reference state). Figure 3 shows that these calculated values match well the values from NIST over the selected variation range (1600–2000 K, 1600–2000 MPa), which supports the correctness of the universal model.

In addition, the expressions for other thermodynamic quantities can be derived under the universal model. For example, the expression for the compressibility factor, *Z*, is obtained based on Eqs. (35), (36) and (37):

41 $$Z = \frac{PV}{{RT}} = \alpha \left( {1 + \frac{1}{\beta }} \right)\frac{{C_{V} }}{R} - \alpha \frac{{C_{\alpha } }}{R}\frac{P}{T}.$$

**Fig. 4:**
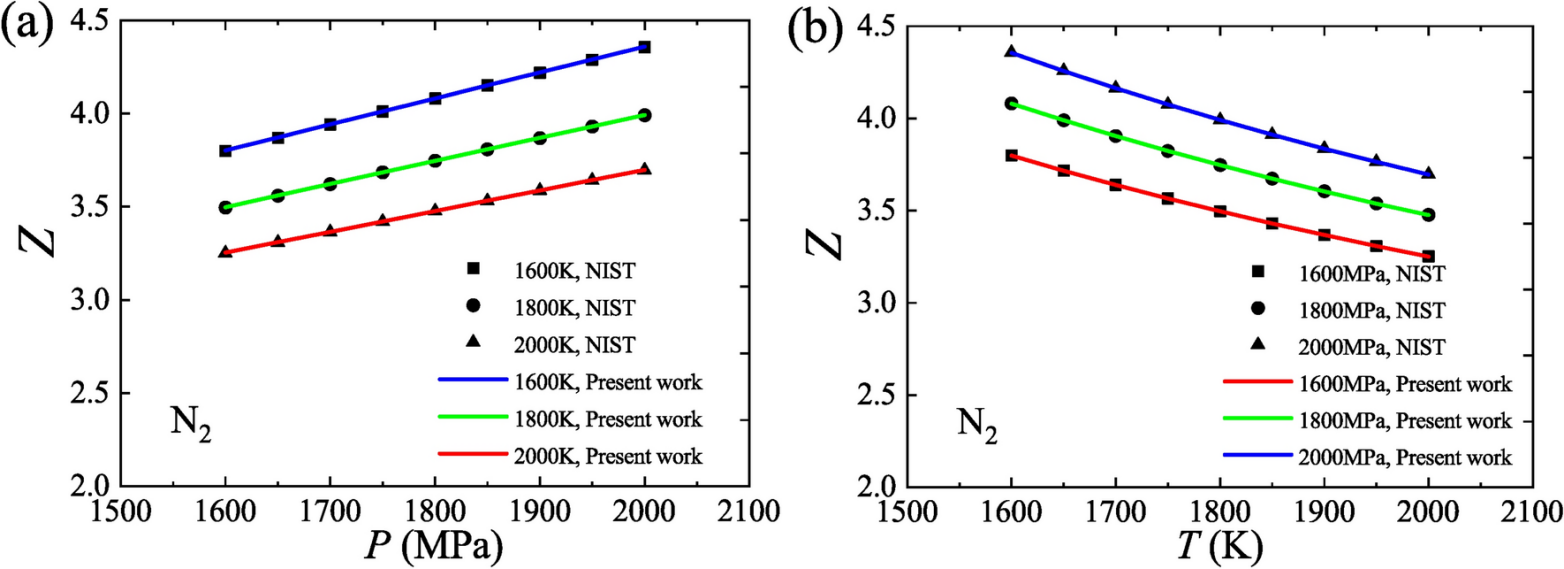
Verification of the compressibility factor using N_2_ data. (**a**) Isotherms, *Z* versus *P*; (**b**) Isobars, *Z* versus *T*.

The universal model tells that the compressibility factor, *Z*, is closely related to the specific heat (capacity) at constant volume, *C*_*V*_, and the ratio of pressure to temperature, *P*/*T*. Based on Eq. (41), the values of *Z* for N_2_ are calculated. Figure 4 shows that these calculated values agree well with the values from NIST.

## Applications

As stated in the introduction, extrapolation based on the ideal gas model performed poorly in characterizing high-density substances. In contrast, the universal EOS has simple form as well as being not restricted by density conditions, so may be advantageous in describing high-density or high-pressure substances.

**Fig. 5:**
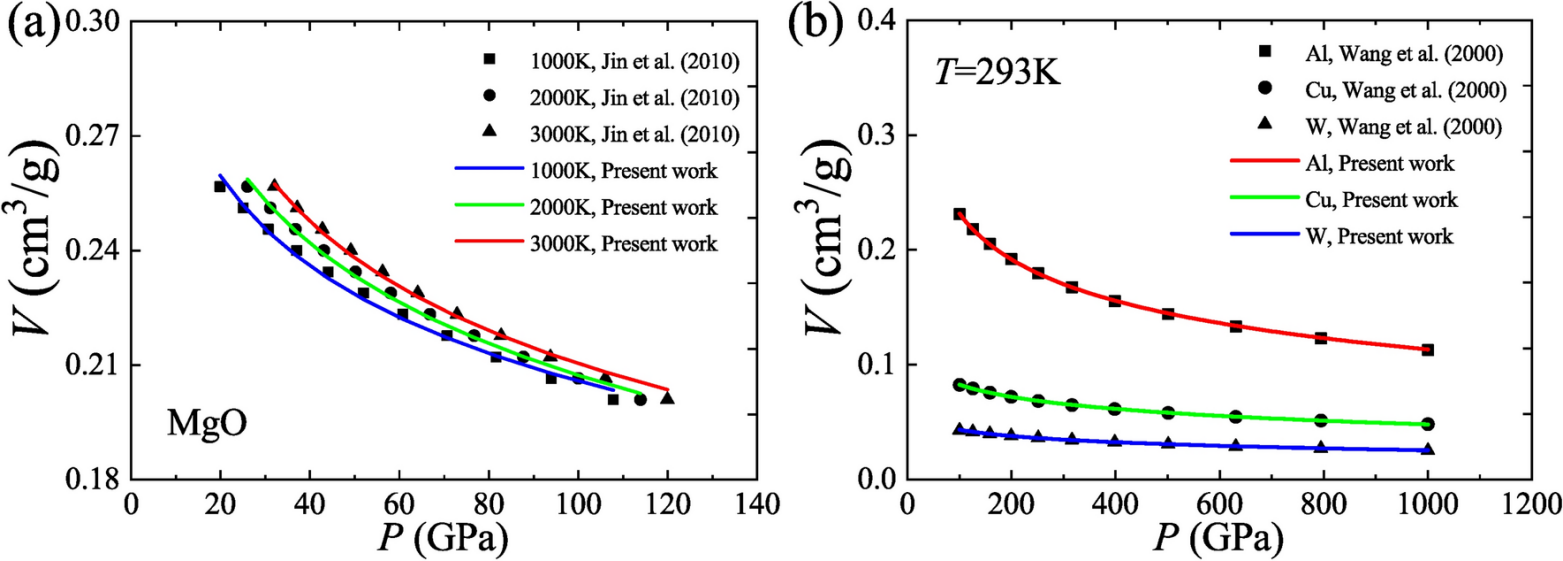
Description of shock Hugoniot data based on the universal *P–V–T* EOS. (**a**) Isotherms for MgO at 1000 K, 2000 K and 3000 K; (**b**) Isotherms for Al, Cu and W at 293 K.

The universal *P–V–T* EOS, Eq. (35), is fitted using the shock Hugoniot data of MgO^33^ at three different temperatures and three metals (Al, Cu and W)^34^ at 293 K respectively. These available data have extremely high pressures (up to 120 GPa for MgO and up to 1000 GPa for Al, Cu and W), which was confirmed to have a relatively high credibility after cross-checking with different experiments as well as various theoretical models^33,34^. They agree well with the universal *P–V–T* EOS, as shown in Fig. 5. The fits have very high R^2^ values (> 0.96).

**Fig. 6:**
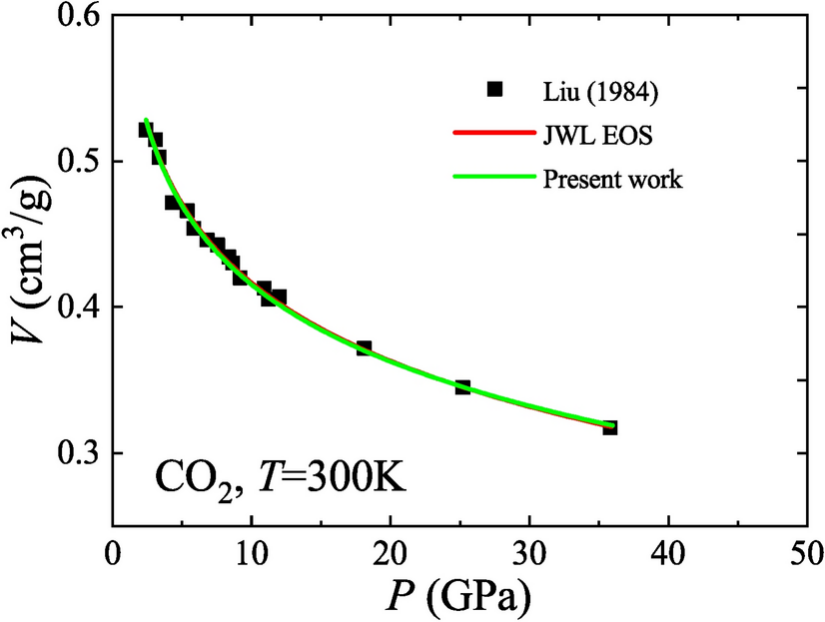
Comparison of the universal *P–V–T* EOS with the JWL EOS using high-pressure CO_2_ data.

The universal model is further compared with the Jones-Wilkins-Lee (JWL) EOS, a well-known empirical EOS in explosive physics. The simplified form of the JWL EOS is as follows^22,35^

42 $$P = A{\text{e}}^{{ - R_{1} V}} { + }\frac{RT}{V},$$

where *A* and *R*_1_ are constants. The JWL EOS is actually a superposition of the ideal gas model and the ideal dense matter model^26^. The first term on the right side of Eq. (42) represents the part of the ideal dense matter model and the second term represents the part of the ideal gas model. As a comparison, the universal model also keeps information from both the ideal gas model and the ideal dense matter model. It will be reduced to the ideal gas model in the low-density limit and be reduced to the ideal dense matter model in the high-density limit. Both the universal *P–V–T* EOS and the JWL EOS are in good agreement with the CO_2_ data from the high-pressure experiment of Liu^36^, as shown in Fig. 6. Yet, as mentioned earlier, the coefficients in the universal *P–V–T* EOS have a clear thermodynamic meaning and can be determined from a priori knowledge, while the coefficients in the JWL EOS are empirical and need to be determined by fitting experimental data.

**Fig. 7:**
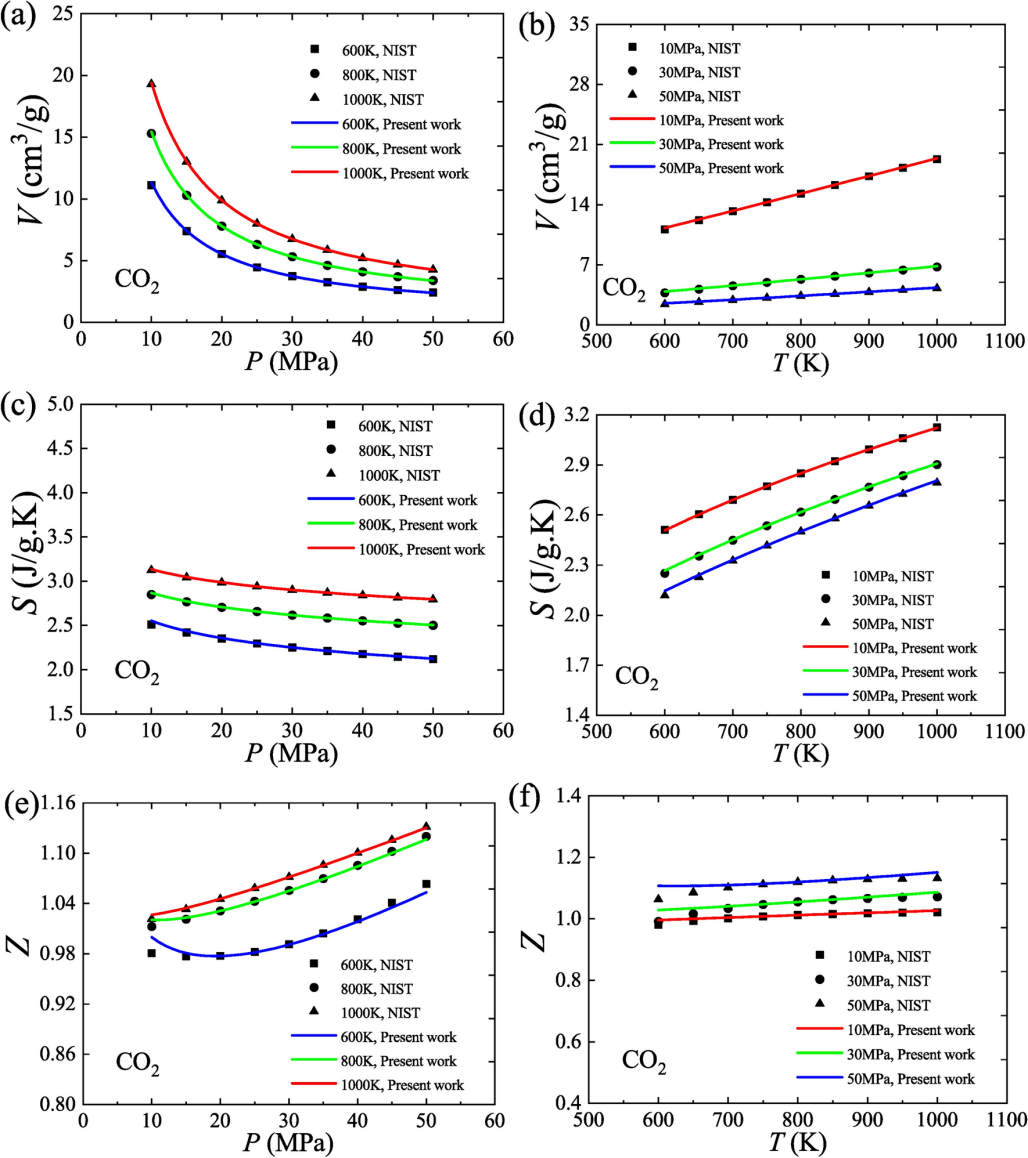
Application of the proposed equation-of-state model to supercritical CO_2_. (**a**) Isotherms, *V* versus *P*; (**b**) Isobars, *V* versus *T*; (**c**) Isotherms, *S* versus *P*; (**d**) Isobars, *S* versus *T*; (**e**) Isotherms, *Z* versus *P*; (**f**) Isobars, *Z* versus *T*.

In addition, CO_2_ has significant industrial applications in its supercritical state. We analyze the property data of supercritical CO_2_ under common operating conditions (600–1000 K, 10–50 MPa) with the proposed model. These data are also from NIST, and their reference point is the saturated liquid state at 273.15 K. The results show that the proposed model agrees well with the property data of volume, entropy and compression factor, as shown in Fig. 7. Besides, Fig. 7e shows that the model can predict the existence of a minimum value of the compression factor as it varies with pressure at 600 K, which is consistent with the compression characteristics of CO_2_.

## Conclusions

Two new dimensionless coefficients of the same family as the Grüneisen coefficient are presented. Analysis shows that both are weak functions of temperature or pressure, and thus can be considered as constants over a certain variation range of matter states. Based on these two dimensionless coefficients, two new thermodynamic functions are further constructed as a single variable function of pressure and as a single variable function of temperature, respectively.

Based on these two single variable functions, a universal EOS model including two forms, *P–V–T* and *P–S–T*, is derived by a purely macroscopic thermodynamic approach. It is universal and covers the commonality of thermodynamic behavior of matter. The forms of these two universal EOS are almost as simple as the ideal gas EOS or the ideal dense matter EOS. It can be demonstrated that the universal model simplifies to the ideal gas model when the state of matter tends to the low-density limit and to the ideal dense matter model when the state of matter tends to the high-density limit.

The coefficients in these two universal EOS have clear thermodynamic meaning and can be determined in advance from other accessible parameters, rather than having to be obtained by experimental fitting. The values of volume, entropy and compressibility factor for N_2_ are calculated directly under the universal model and closely match the data from NIST. In addition, the universal model is shown to have a clear advantage in high-pressure applied sciences such as the shock-Hugoniots and also have important applications in engineering practices involving supercritical CO_2_.

## Supplementary Information

Supplementary Information.

## Data Availability

The data that support the findings of this study are available from the corresponding author upon reasonable request.

## List of symbols

*P*: Pressure
*T*: Temperature
*V*: Volume
*S*: Entropy
*R*: Ideal gas constant
*R*′: Ideal dense matter constant
*C*_*V*_: Specific heat at constant volume
*C*_*S*_: Specific work at constant entropy
*U*: Internal energy
*Z*: Compressibility factor
*γ*: Grüneisen coefficient
*E*_*γ*_: Thermodynamic function related to *γ*
*α*: Dimensionless coefficient
*E*_*α*_: Thermodynamic function related to *α*
*β*: Dimensionless coefficient
*E*_*β*_: Thermodynamic function related to *β*
*α*_*P*_: Isobaric expansion coefficient
*β*_*T*_: Isothermal compression coefficient
*C*_*α*_: Work capacity corresponding to* E*_*α*_
*C*_*β*_: Heat capacity corresponding to *E*_*β*_

## References

[1] Cengel, Y. A. & Boles, M. A. *Thermodynamics: An Engineering Approach* (McGraw-Hill, 2006).

[2] van der Waals, J. D. *Over de Continuiteit van den Gasen Vloeistoftoestand.* (Doctoral Dissertation, Leiden: Leiden University, 1873).

[3] Klein, M. J. The historical origins of the van der Waals equation. *Physica* **73**, 28 (1974).

[4] Sengers, J. V., Kayser, R. F., Peters, C. J. & White, H. J. *Equations of State for Fluids and Fluid Mixtures* (Elsevier, 2000).

[5] Redlich, O. & Kwong, J. N. On the thermodynamics of solutions. V. An equation of state. Fugacities of gaseous solutions. *Chem. Rev.* **44**(1), 233–244 (1949). 18125401 10.1021/cr60137a013

[6] Peng, D. Y. & Robinson, D. B. A new two-constant equation of state. *Ind. Eng. Chem. Fundam.* **15**(1), 59–64 (1976).

[7] Benedict, M., Webb, G. B. & Rubin, L. C. An empirical equation for thermodynamic properties of light hydrocarbons and their mixtures I. Methane, ethane, propane and n-butane. *J. Chem. Phys.* **8**, 334 (1940).

[8] Benedict, M., Webb, G. B. & Rubin, L. C. An empirical equation for thermodynamic properties of light hydrocarbons and their mixtures II. Mixtures of methane, ethane, propane, and n-butane. *J. Chem. Phys.* **10**, 747 (1942).

[9] Postnikov, E. B., Goncharov, A. L. & Melent’ev, V. V. Tait equation revisited from the entropic and fluctuational points of view. *Int. J. Thermophys.* **35**, 2115 (2014).

[10] Sun, J. X., Wu, Q., Guo, Y. & Cai, L. C. Two universal equations of state for solids. *Zeitschrift für Naturforschung A* **65**(1–2), 34–44 (2010).

[11] Heuzé, O. General form of the Mie-Grüneisen equation of state. *Comptes Rendus Mécanique* **340**(10), 679–687 (2012).

[12] Drebushchak, V. A. Thermal expansion of solids: Review on theories. *J. Therm. Anal. Calorim.* **142**, 1097 (2020).

[13] Ghatak, A. K. & Eliezer, S. A review on equations of state at high densities based on Thomas-Fermi and related models. *Laser Part Beams* **2**, 309 (1984).

[14] Baker, G. A. Jr. & Johnson, J. D. General structure of the Thomas-Fermi equation of state. *Phys. Rev. A* **44**, 2271 (1991). 9906209 10.1103/physreva.44.2271

[15] Parsafar, G. A., Spohr, H. V. & Patey, G. N. An accurate equation of state for fluids and solids. *J. Phys. Chem. B* **113**, 11977 (2009). 19678647 10.1021/jp903519c

[16] Oh, K. H. & Persson, P. A. Equation of state for extrapolation of high-pressure shock Hugoniot data. *J. Appl. Phys.* **65**, 3852 (1989).

[17] Span, R. & Wagner, W. On the extrapolation behavior of empirical equations of state. *Int. J. Thermophys.* **18**, 1415 (1997).

[18] Brosh, E., Shneck, R. Z. & Makov, G. Explicit Gibbs free energy equation of state for solids. *J. Phys. Chem. Solids* **69**, 1912 (2008).

[19] Latimer, K., Dwaraknath, S., Mathew, K., Winston, D. & Persson, K. A. Evaluation of thermodynamic equations of state across chemistry and structure in the materials project. *Npj Comput. Mater.* **4**, 1 (2018).

[20] Nowak, P., Kleinrahm, R. & Wagner, W. Measurement and correlation of the (p, ρ, T) relation of nitrogen. I. The homogeneous gas and liquid regions in the temperature range from 66 K to 340 K at pressures up to 12 MPa. *J. Chem. Thermodyn.* **29**, 1137 (1997).

[21] Crain, R. W. Jr. & Sonntag, R. E. Nitrogen constants for the Benedict-Webb-Rubin equation of state. *J. Chem. Eng. Data* **12**, 73 (1967).

[22] Lee, E. L., Hornig, H. C. & Kury, J. W. *Adiabatic Expansion of High Explosive Detonation Products* (No. UCRL-50422). (Univ. of California Radiation Lab. at Livermore, Livermore, CA, United States, 1968).

[23] MacDonald, J. R. Review of some experimental and analytical equations of state. *Rev. Mod. Phys.* **41**, 316 (1969).

[24] Xue, T. W. & Guo, Z. Y. A general equation of state for high density matter from thermodynamic symmetry. *J. Appl. Phys.* **131**, 044902 (2022).

[25] Li, Y., Xue, T. W., Su, C. J. & Guo, Z. Y. Verification of ideal dense matter equation of state by molecular dynamics simulation. *J. Appl. Phys.* **132**, 165901 (2022).

[26] Xue, T. W. & Guo, Z. Y. A global equation-of-state model from mathematical interpolation between low-and high-density limits. *Sci. Rep.* **12**, 12533 (2022). 35869101 10.1038/s41598-022-16016-6 PMC9307579

[27] Bordoni, S. Routes towards an abstract thermodynamics in the late nineteenth century. *Eur. Phys. J. H* **38**(5), 617 (2013).

[28] Helm, G. *The Historical Development of Energetics* (Springer, 2013).

[29] von Oettingen, A. J. Die thermodynamischen Beziehungen antithetisch entwickelt. *Mem. Acad. Sci. St. Petersb.* **32**, 1–70 (1885).

[30] Barron, T. H. K. Grüneisen parameters for the equation of state of solids. *Ann. Phys.* **1**(1), 77–90 (1957).

[31] Burakovsky, L. & Preston, D. L. Analytic model of the Grüneisen parameter all densities. *J. Phys. Chem. Solids* **65**(8–9), 1581–1587 (2004).

[32] Santoro, M. et al. Amorphous silica-like carbon dioxide. *Nature* **441**, 857–860 (2006). 16778885 10.1038/nature04879

[33] Jin, K. et al. The pressure-volume-temperature equation of state of MgO derived from shock Hugoniot data and its application as a pressure scale. *J. Appl. Phys.* **107**, 113518 (2010).

[34] Wang, Y., Chen, D. & Zhang, X. Calculated equation of state of Al, Cu, Ta, Mo, and W to 1000 GPa. *Phys. Rev. Lett.* **84**(15), 3220 (2000). 11019055 10.1103/PhysRevLett.84.3220

[35] Baudin, G. & Serradeill, R. Review of Jones-Wilkins-Lee equation of state. In *EPJ Web of Conferences* Vol. 10, p. 00021. (EDP Sciences, 2010).

[36] Liu, L. G. Compression and phase behavior of solid CO_2_ to half a megabar. *Earth Planet Sci. Lett.* **71**, 104 (1984).

