# Supplementary material for: A universal equation-of-state model based on single variable functions
Source: Sci Rep. 2025 Mar 12;15:8475. doi: 10.1038/s41598-025-93314-9 (PMC11897379; doi:10.1038/s41598-025-93314-9)
Supplement: Supplementary file 1 — Supplementary Information. [file 41598_2025_93314_MOESM1_ESM.docx]

**A universal equation-of-state model based on single variable functions**

**Ti-Wei Xue and Zeng-Yuan Guo***

Key Laboratory for Thermal Science and Power Engineering of Ministry of Education, Department of Engineering Mechanics, Tsinghua University, Beijing 100084, China

***** Correspondence: demgzy@tsinghua.edu.cn

**Thermodynamic derivation of equations of state**

Combining Eqs. (17), (18), (20) and (21) in the text yields

*$\frac{1}{\alpha}\text{d}\left[\left(V+\alpha C_{\alpha}\right)P\right]=-\frac{1}{\beta}\text{d}\left[\left(S-\beta C_{\beta}\right)T\right]$*. (S1)

With pressure and temperature as independent variables, taking partial derivatives with respect to pressure for each term in Eq. (S1) yields

*$\frac{1}{\alpha}\left(V+\alpha C_{\alpha}\right)\text{+}\frac{1}{\alpha}P\left[\frac{\partial\left(V+\alpha C_{\alpha}\right)}{\partial P}\right]_{T}=-\frac{1}{\beta}T\left[\frac{\partial\left(S-\beta C_{\beta}\right)}{\partial P}\right]_{T}$*. (S2)

Since *α*, *β*, *C_α_* and *C_β_* are treated as constants, the Maxwell relation corresponding to the Gibbs free energy can be written as

*$\left[\frac{\partial\left(V+\alpha C_{\alpha}\right)}{\partial T}\right]_{P}=-\left[\frac{\partial\left(S-\beta C_{\beta}\right)}{\partial P}\right]_{T}$*. (S3)

Substituting Eq. (S3) into Eq. (S2) gives

*$\frac{1}{\alpha}\left(V+\alpha C_{\alpha}\right)\text{+}\frac{1}{\alpha}P\left[\frac{\partial\left(V+\alpha C_{\alpha}\right)}{\partial P}\right]_{T}=\frac{1}{\beta}T\left[\frac{\partial\left(V+\alpha C_{\alpha}\right)}{\partial T}\right]_{P}$*. (S4)

There is the following thermodynamic cyclic relation that holds forever:

*$\left[\frac{\partial\left(V+\alpha C_{\alpha}\right)}{\partial T}\right]_{P}\text{=}-\left(\frac{\partial P}{\partial T}\right)_{V}\left[\frac{\partial\left(V+\alpha C_{\alpha}\right)}{\partial P}\right]_{T}$*. (S5)

Substituting Eq. (S5) into Eq. (S4) gives

*$\frac{1}{\alpha}\left(V+\alpha C_{\alpha}\right)\text{+}\frac{1}{\alpha}P\left[\frac{\partial\left(V+\alpha C_{\alpha}\right)}{\partial P}\right]_{T}=-\frac{1}{\beta}T\left(\frac{\partial P}{\partial T}\right)_{V}\left[\frac{\partial\left(V+\alpha C_{\alpha}\right)}{\partial P}\right]_{T}$*. (S6)

With volume and temperature as independent variables, taking partial derivative of *E_β_* with respect to volume yields

*$\left(\frac{\partial S}{\partial V}\right)_{T}=\frac{1}{1\text{+}\frac{1}{\beta}}\frac{P}{T}$*. (S7)

There is the Maxwell relation corresponding to the Helmholtz free energy:

*$\left(\frac{\partial P}{\partial T}\right)_{V}\text{=}\left(\frac{\partial S}{\partial V}\right)_{T}$*. (S8)

Combining Eqs. (S6), (S7) and (S8) yields

*$\frac{V+\alpha C_{\alpha}}{P}=-\left(\frac{1+\alpha\text{+}\beta}{1\text{+}\beta}\right)\left[\frac{\partial\left(V+\alpha C_{\alpha}\right)}{\partial P}\right]_{T}$*. (S9)

Integrating Eq. (S9) yields

*$V=f\left(T\right)P^{-\frac{1\text{+}\beta}{1+\alpha\text{+}\beta}}-\alpha C_{\alpha}$*, (S10)

where *f* (*T*) is an unknown function of temperature, whose expression needs to be determined. Substituting Eq. (S5) again into Eq. (S4) yields another form:

*$\frac{1}{\alpha}\left(V+\alpha C_{\alpha}\right)-\frac{1}{\alpha}P\left(\frac{\partial T}{\partial P}\right)_{V}\left[\frac{\partial\left(V+\alpha C_{\alpha}\right)}{\partial T}\right]_{P}=\frac{1}{\beta}T\left[\frac{\partial\left(V+\alpha C_{\alpha}\right)}{\partial T}\right]_{P}$*. (S11)

Combining Eqs. (S7), (S8) and (S11) yields

*$\frac{V+\alpha C_{\alpha}}{T}=\left(\frac{1+\alpha+\beta}{\beta}\right)\left[\frac{\partial\left(V+\alpha C_{\alpha}\right)}{\partial T}\right]_{P}$*. (S12)

Integrating Eq. (S12) yields

*$V=f\left(P\right)T^{\frac{\beta}{1+\alpha\text{+}\beta}}-\alpha C_{\alpha}$*. (S13)

Combining Eqs. (S10) and (S13) yields

*$f\left(T\right)P^{-\frac{1\text{+}\beta}{1+\alpha\text{+}\beta}}=f\left(P\right)T^{\frac{\beta}{1+\alpha\text{+}\beta}}$*. (S14)

Rewrite Eq. (S14) as

*$f\left(T\right)T^{-\frac{\beta}{1+\alpha\text{+}\beta}}=f\left(P\right)P^{\frac{1\text{+}\beta}{1+\alpha\text{+}\beta}}$*. (S15)

Since the left side of the equal sign in Eq. (S15) is a function of temperature only and the right side is a function of pressure only, they can be equal to the same constant only. Thus, the two unknown functions, *f* (*T*) and *f* (*P*), are determined as

*$f\left(T\right)=C_{1}T^{\frac{\beta}{1+\alpha\text{+}\beta}}$*, (S16)

*$f\left(P\right)=C_{1}P^{-\frac{1\text{+}\beta}{1+\alpha\text{+}\beta}}$*, (S17)

where *C*_1_ is the mentioned constant. Substituting Eq. (S16) into Eq. (S10) yields the *P-V-T* equation of state (EOS):

*$V=C_{1}T^{\frac{\beta}{1+\alpha\text{+}\beta}}P^{-\frac{1\text{+}\beta}{1+\alpha\text{+}\beta}}-\alpha C_{\alpha}$*. (S18)

Of course, substituting Eq. (S17) into Eq. (S13) also yields Eq. (S18).

The same approach is used to derive the *P-S-T* EOS. With pressure and temperature as independent variables, taking partial derivatives with respect to temperature for each term in Eq. (S1) yields

*$\frac{1}{\alpha}P\left[\frac{\partial\left(V+\alpha C_{\alpha}\right)}{\partial T}\right]_{P}=-\frac{1}{\beta}\left(S-\beta C_{\beta}\right)-\frac{1}{\beta}T\left[\frac{\partial\left(S-\beta C_{\beta}\right)}{\partial T}\right]_{P}$*. (S19)

Substituting Eq. (S3) into Eq. (S19) gives

*$\frac{1}{\alpha}P\left[\frac{\partial\left(S-\beta C_{\beta}\right)}{\partial P}\right]_{T}=\frac{1}{\beta}\left(S-\beta C_{\beta}\right)\text{+}\frac{1}{\beta}T\left[\frac{\partial\left(S-\beta C_{\beta}\right)}{\partial T}\right]_{P}$*. (S20)

There is the following thermodynamic cyclic relation that holds forever:

*$\left[\frac{\partial\left(S-\beta C_{\beta}\right)}{\partial P}\right]_{T}\text{=}-\left(\frac{\partial T}{\partial P}\right)_{S}\left[\frac{\partial\left(S-\beta C_{\beta}\right)}{\partial T}\right]_{P}$*. (S21)

Substituting Eq. (S21) into Eq. (S20) gives

*$\frac{1}{\alpha}P\left(\frac{\partial T}{\partial P}\right)_{S}\left[\frac{\partial\left(S-\beta C_{\beta}\right)}{\partial T}\right]_{P}=-\frac{1}{\beta}\left(S-\beta C_{\beta}\right)-\frac{1}{\beta}T\left[\frac{\partial\left(S-\beta C_{\beta}\right)}{\partial T}\right]_{P}$*. (S22)

With entropy and pressure as independent variables, taking partial derivative of *E_α_* with respect to entropy yields

*$\left(\frac{\partial V}{\partial S}\right)_{P}=\frac{1}{1+\frac{1}{\alpha}}\frac{T}{P}$*. (S23)

There is the Maxwell relation corresponding to enthalpy:

*$\left(\frac{\partial T}{\partial P}\right)_{S}\text{=}\left(\frac{\partial V}{\partial S}\right)_{P}$*. (S24)

Combining Eqs. (S22), (S23) and (S24) yields

*$\frac{S-\beta C_{\beta}}{T}=-\frac{1+\alpha+\beta}{1+\alpha}\left[\frac{\partial\left(S-\beta C_{\beta}\right)}{\partial T}\right]_{P}$*. (S25)

Integrating Eq. (S25) yields

*$S=f\left(P\right)T^{-\frac{1+\alpha}{1\text{+}\alpha+\beta}}+\beta C_{\beta}$*, (S26)

where *f* (*P*) is an unknown function of pressure, whose expression needs to be determined. Substituting Eq. (S21) again into Eq. (S20) yields another form:

*$\frac{1}{\alpha}P\left[\frac{\partial\left(S-\beta C_{\beta}\right)}{\partial P}\right]_{T}=\frac{1}{\beta}\left(S-\beta C_{\beta}\right)-\frac{1}{\beta}T\left(\frac{\partial P}{\partial T}\right)_{S}\left[\frac{\partial\left(S-\beta C_{\beta}\right)}{\partial P}\right]_{T}$*. (S27)

Combining Eqs. (S23), (S24) and (S27) yields

*$\frac{S-\beta C_{\beta}}{P}=\left(\frac{1+\alpha+\beta}{\alpha}\right)\left[\frac{\partial\left(S-\beta C_{\beta}\right)}{\partial P}\right]_{T}$*. (S28)

Integrating Eq. (S28) yields

*$S=f\left(T\right)P^{\frac{\alpha}{1\text{+}\alpha\text{+}\beta}}\text{+}\beta C_{\beta}$*. (S29)

Combining Eqs. (S26) and (S29) with the same mathematical technique as before yields the *P-S-T* EOS:

*$S=C_{2}T^{-\frac{1+\alpha}{1\text{+}\alpha+\beta}}P^{\frac{\alpha}{1\text{+}\alpha\text{+}\beta}}\text{+}\beta C_{\beta}$*, (S30)

where *C*_2_ is another constant.

Take the partial derivatives of Eqs. (S18) and (S30), respectively and substitute them into the Maxwell relation, Eq. (S3). Then the relation between the two constants, *C*_1_ and *C*_2_, is obtained:

*$\frac{C_{1}}{\alpha}=-\frac{C_{2}}{\beta}$*. (S31)

Making each term on both sides of the equal sign of Eq. (S31) equal to the same constant, *C*, the two EOS can be rewritten as

*$V=\alpha CT^{\frac{\beta}{1+\alpha\text{+}\beta}}P^{-\frac{1\text{+}\beta}{1+\alpha\text{+}\beta}}-\alpha C_{\alpha}$*, (S32)

*$S=-\beta CT^{-\frac{1+\alpha}{1\text{+}\alpha+\beta}}P^{\frac{\alpha}{1\text{+}\alpha\text{+}\beta}}\text{+}\beta C_{\beta}$*. (S33)

Eqs. (S32) and (S33) are Eqs. (23) and (24) in the text, respectively.
